# Automated Detection of Anatomical Landmarks During Colonoscopy Using a Deep Learning Model

**DOI:** 10.1093/jcag/gwad017

**Published:** 2023-05-02

**Authors:** Mahsa Taghiakbari, Sina Hamidi Ghalehjegh, Emmanuel Jehanno, Tess Berthier, Lisa di Jorio, Saber Ghadakzadeh, Alan Barkun, Mark Takla, Mickael Bouin, Eric Deslandres, Simon Bouchard, Sacha Sidani, Yoshua Bengio, Daniel von Renteln

**Affiliations:** Faculty of Medicine, Department of Biomedical Sciences, University of Montreal, Montreal, Quebec, Canada; Department of Medicine, Division of Gastroenterology, University of Montreal Hospital Research Center (CRCHUM), Montreal, Quebec, Canada; Department of Artificial Intelligence, Imagia Canexia Health Inc., Montreal, Canada; Department of Artificial Intelligence, Imagia Canexia Health Inc., Montreal, Canada; Department of Artificial Intelligence, Imagia Canexia Health Inc., Montreal, Canada; Department of Artificial Intelligence, Imagia Canexia Health Inc., Montreal, Canada; Department of Artificial Intelligence, Imagia Canexia Health Inc., Montreal, Canada; Division of Gastroenterology, McGill University Health Center, McGill University, Montreal, Quebec, Canada; Faculty of Medicine, Department of Biomedical Sciences, University of Montreal, Montreal, Quebec, Canada; Department of Medicine, Division of Gastroenterology, University of Montreal Hospital Research Center (CRCHUM), Montreal, Quebec, Canada; Department of Medicine, Division of Gastroenterology, University of Montreal Hospital Research Center (CRCHUM), Montreal, Quebec, Canada; Division of Gastroenterology, University of Montreal Hospital Center (CHUM), Montreal, Quebec, Canada; Division of Gastroenterology, University of Montreal Hospital Center (CHUM), Montreal, Quebec, Canada; Division of Gastroenterology, University of Montreal Hospital Center (CHUM), Montreal, Quebec, Canada; Division of Gastroenterology, University of Montreal Hospital Center (CHUM), Montreal, Quebec, Canada; Faculty of Medicine, Department of Biomedical Sciences, University of Montreal, Montreal, Quebec, Canada; Department of Medicine, Division of Gastroenterology, University of Montreal Hospital Research Center (CRCHUM), Montreal, Quebec, Canada; Division of Gastroenterology, University of Montreal Hospital Center (CHUM), Montreal, Quebec, Canada

**Keywords:** Artificial intelligence, Colonoscopy, Colorectal polyp, Deep learning, Endoscopy, Ileocecal valve

## Abstract

**Background and aims:**

Identification and photo-documentation of the ileocecal valve (ICV) and appendiceal orifice (AO) confirm completeness of colonoscopy examinations. We aimed to develop and test a deep convolutional neural network (DCNN) model that can automatically identify ICV and AO, and differentiate these landmarks from normal mucosa and colorectal polyps.

**Methods:**

We prospectively collected annotated full-length colonoscopy videos of 318 patients undergoing outpatient colonoscopies. We created three nonoverlapping training, validation, and test data sets with 25,444 unaltered frames extracted from the colonoscopy videos showing four landmarks/image classes (AO, ICV, normal mucosa, and polyps). A DCNN classification model was developed, validated, and tested in separate data sets of images containing the four different landmarks.

**Results:**

After training and validation, the DCNN model could identify both AO and ICV in 18 out of 21 patients (85.7%). The accuracy of the model for differentiating AO from normal mucosa, and ICV from normal mucosa were 86.4% (95% CI 84.1% to 88.5%), and 86.4% (95% CI 84.1% to 88.6%), respectively. Furthermore, the accuracy of the model for differentiating polyps from normal mucosa was 88.6% (95% CI 86.6% to 90.3%).

**Conclusion:**

This model offers a novel tool to assist endoscopists with automated identification of AO and ICV during colonoscopy. The model can reliably distinguish these anatomical landmarks from normal mucosa and colorectal polyps. It can be implemented into automated colonoscopy report generation, photo-documentation, and quality auditing solutions to improve colonoscopy reporting quality.

## INTRODUCTION

Colonoscopy is a key component of effective colorectal cancer (CRC) prevention programs (1,2). A high-quality colonoscopy is achieved through a complete examination that results in a high adenoma detection rate (ADR), which reduces the risk of patients developing interval CRC (3–5). As colonoscopy is operator dependent, multiple gastroenterology initiatives have recommended that endoscopists achieve minimum performance scores. This is represented through a cecal intubation rate (CIR) of >90% (3). In order to demonstrate cecal intubation and completeness of the examination, current guidelines request identification and photo-documentation of the ileocecal valve (ICV) and appendiceal orifice (AO) (3,6). Recent advancements in artificial intelligence (AI) and the development of the deep convolutional neural network (DCNN) allow for real-time image processing during colonoscopy. This enables automatic detection of anatomical structures during live endoscopies. To date, AI has mainly assisted endoscopists in the detection and classification of colorectal polyps (7–9). We hypothesized that an AI-empowered solution could help us automatically differentiate anatomical landmarks such as AO and ICV from polyps and normal colon mucosa. Such an AI solution could be incorporated into colonoscopy report-generating software, help with automated photo-documentation, or be used for quality auditing. Therefore, we conducted a study developing a DCNN-based model to differentiate the AO, ICV, and polyps from normal colon mucosa, and to confirm automated detection of AO and ICV in a test set.

## METHODS

### Study Population

We prospectively enrolled 358 consecutive patients aged 45 to 80 years who attended the Centre Hospitalier de l’Université de Montréal (CHUM) for an elective colonoscopy between January and October 2021. Exclusion criteria were explained in the Supplementary File. Additionally, colonoscopy videos in which technical failures led to problems recording the colonoscopy procedure were also excluded (n = 17). Thus, colonoscopy videos from 318 patients were included in the final analyses. All included patients signed informed consents for study participation, video recording, and further analyses of the videos. The study protocol was approved by the local ethics board (IRB #: 20.198) and was registered at https://clinicaltrials.gov/ (NCT04586556).

### Study Procedure

All colonoscopies were performed by five board-certified gastroenterologists according to the current standard of care using standard high-definition colonoscopes (Olympus 190 series; Olympus Corp., Center Valley, PA, USA) (3). The colonoscopy videos were recorded using Medicapture USB 300 devices (high definition, 1080, H.264/MPEG4) and stored on a hard drive. The endoscopists were instructed to use narrow-band imaging for performing optical diagnosis at their discretion. Endoscopists removed detected polyps using standard polypectomy techniques, and the specimens were sent to the local histopathology laboratory for histology assessment. All patients were followed up after 2 weeks to inquire about delayed adverse events. No severe adverse events were reported. All videos were deidentified by removing any patient identifier information before being permanently stored on a local hard drive. A research assistant attended each colonoscopy procedure to document all relevant study steps on standardized case report forms. The research assistant started a stopwatch function upon colonoscope insertion into the rectum to enable documentation of the exact withdrawal time and moment of landmark detection in order to create annotated video files.

Based on the recommendation of the Canadian Association of Gastroenterology (10) for standard colonoscopy procedures, the following data were collected. (a) Patient demographic and clinical characteristics, including age, sex, body mass index, family history of CRC, colonoscopy indication, and ASA classification. (b) General procedural data, including date and time of the procedure and the endoscopist’s name. (c) Colonoscopy characteristics, including bowel preparation quality (poor vs. adequate, defined as an overall BBPS score >6, and >2 for each colon segment (11)), the exact time of colonoscope insertion in the rectum, the exact time of identifying important anatomical landmarks (i.e., AO, ICV), cecal intubation (as a surrogate for complete colonoscopy, yes/no), the exact time of starting withdrawal of the colonoscope, the exact time the colonoscope reached and was removed from the rectum, and withdrawal time (defined as the time required to withdraw the colonoscope from cecal intubation to removal from the anus). (d) Polyp-related characteristics, including the exact time of detection of each polyp (if multiple), and anatomical location, size, and morphology (according to the Paris classification (12), polypoid/nonpolypoid) of each polyp. We dedicated a specific code to each endoscope and patient to avoid confusion. Therefore, all collected data on the case report forms were anonymized before being transferred to an electronic database.

### Model Training and Validation

**Figure 1.**
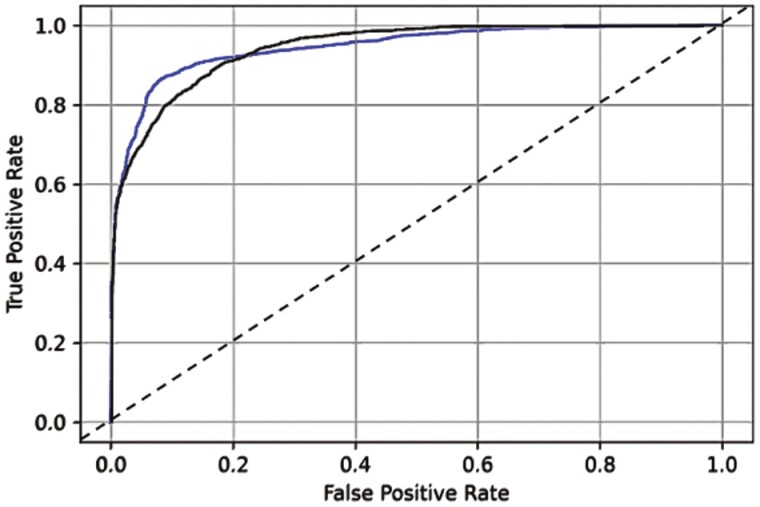
The area under the curve of the deep convolutional neural network model to distinguish appendiceal orifice versus ileocecal valve, and versus normal mucosa (blue line; AUC = 94.5 [95% CI 93.8 to 95.3]), and the appendiceal orifice versus ileocecal valve versus polyp, and versus normal mucosa (black line; AUC = 94.4 [95% CI 93.9 to 94.9]). The black dashed line represents the reference line.

We trained a DCNN AI model on 21,503 unaltered frames extracted from the recorded colonoscopy videos of 272 patients, and validated and tested the model on 1924 (25 patients) and 2017 (21 patients) unaltered frames, respectively. Supplementary Table 1 shows the detailed patient demographic and procedural characteristics used in each data set. All frames were extracted from the white-light colonoscopies, and all narrow-band imaging frames were excluded. We followed the procedure shown in Figure 1 to extract the required frames for training and testing the AI model. The model was trained to distinguish between four distinct landmarks: (a) AO, (b) ICV, (c) polyp, and (d) normal mucosa. For each landmark, we extracted an average of 30 frames for each time of its appearance. As consecutive frames within a video are correlated, we introduced a stride of 4 frames (i.e., the amount of movement over the frames of a video) for the AO, ICV, and polyp landmarks, and a random stride of between 4 and 15 frames for the normal mucosa landmark during the frame extraction. This was to increase the exposure of the model to higher variability among nonconsecutive frames.

As the annotation for timing of landmark detection in real-time might not be precise, there was a possibility that some of the extracted frames would not contain their corresponding landmarks. Furthermore, because of the movement of the colonoscope inside the colon, sometimes the landmark of interest might disappear from the field of view for a short period of time. Therefore, to ensure that we used labeled frames for model training correctly, all the extracted frames were reviewed and annotated by a team of three clinicians (MT, MT, DvR). Using a quality assessment tool, the clinicians examined a total of 86,754 frames (7982 AO, 8374 ICV, 32,971 polyps, and 37,427 normal mucosa) and verified whether or not the frame contained one unique landmark. If a frame was too blurry or contained two landmarks, or a very small portion of a landmark from which even an expert clinician could not locate the object, the frame was discarded. After performing the verification process, 25,444 frames (2914 AO, 2606 ICV, 14,772 polyps, and 5152 normal mucosa) were accepted to be used for model training, validation, and testing (Table 1). The training, validation, and test data sets did not overlap (details provided in Supplementary Table 1).

**Table 1.** Number of frames used for artificial intelligence model training, validation, and testing

Number of Frames	Total	Rejected frames	Frames not tagged	Accepted frames	Number of frames used in training data set	Number of frames used in validation data set	Number of frames used in test data set
Normal mucosa	37,427	5172	27,103	5,152	4,103	519	530
Polyp^*^	32,971	17,353	846	14,772	13,479	651	642
Ileocecal valve	8,374	5,619	149	2,606	1,892	322	392
Appendiceal orifice	7,982	4,708	360	2,914	2,029	432	453
Total	86,754	32,852	28,458	25,444	21,503	1,924	2,017

^*^All frames containing polyps were retrieved from white-light colonoscopy videos.

### DCNN-Based AI Model

The DCNN model used in the current study is an off-the-shelf network based on the Inception V3 architecture (13) and pretrained on the ImageNet data set (14). We applied a transfer learning technique to fine-tune the model parameters to the endoscopic images using a cross-entropy loss function and back-propagation algorithm (15). The model was trained to distinguish between AO, ICV, polyp, and normal mucosa. The images associated with different classes were fed to the model in equal proportions to keep the balance across the four classes during the training phase. For all experiments, we used an Adam optimizer with a learning rate of 0.0002. We used a learning rate scheduler with patience of 5 and a factor of 0.5 to decrease the learning rate when the validation accuracy stopped improving. Because of the small volume of data available, different techniques were used to decrease the overfitting of the model, such as different data-augmentation techniques, which were applied to each frame, thus introducing more variability and richer diversity to the model (16). This included 90% to 100% horizontal and vertical scaling, 0-to-5-degree rotation, –5% to 5% horizontal and vertical translation, 95% to 105% colour saturation adjustment, 95% to 105% colour brightness adjustment, random horizontal and vertical flipping, –3% to 3% horizontal and vertical shearing, 0% to 1% perspective, and 0% to 2% sharpening. We used L2 regularization with a penalty of 0.001, a drop-out before the Softmax layer with a drop rate of 0.8, and an early-stopping technique. The model training, validation, and testing were performed using an NVIDIA Tesla V100 GPU with 32 GB of memory.

### Study Outcomes

The primary outcome was the proportion of patients in whom the AI model could identify both ICV and AO, and differentiate them from polyps and normal mucosa, with an accuracy of detecting both AO and ICV above a threshold of 40% (representing a value in which reliable identification of the landmarks can be assumed without increasing false-positive alerts). The secondary outcome was the accuracy of the AI model in differentiating AO (vs. normal mucosa) compared with frames annotated by expert endoscopists, which were used as the reference. Other outcomes included: (a) the accuracy of the AI model to differentiate ICV (vs. normal mucosa) compared with the expert-annotated frames; (b) the accuracy of the AI model to differentiate polyp (vs. normal mucosa); (c) the accuracy of the AI model to differentiate normal mucosa, defined as the colonoscopy images containing no other landmarks (i.e., OA, ICV, polyp, diverticulum); (d) the accuracy of the model to differentiate between AO, ICV, polyp, and normal mucosa when >1 landmark appeared in an image; (e) other diagnostic characteristics of the AI model for differentiating each landmark mentioned above, including sensitivity, specificity, negative and positive predictive values, and the area under the receiver operating characteristic curve (AUC); (f) the false-positive detection rate for each landmark.

### Statistical Analysis

All confidence intervals were computed using Clopper–Pearson interval method for calculating binomial confidence intervals using the extracted confusion matrices from the model that categorized the predictions of each landmark in each image against the actual annotated images in the test data set. The R programming language (R Core Team, 2020) was used for statistical computing of all diagnostic performance values and confidence intervals.

## RESULTS

A total of 2017 frames were used to test the performance of the AI model on unseen data (Table 1). Both AO and ICV could concomitantly be detected in 18 out of 21 patients (85.7%; 95% CI 63.7% to 97.0%) if accuracies were above the threshold of 40%. Table 2 shows details of the codetection of both AO and ICV by the AI model.

**Table 2.** The proportion of patients in the test data set, in which the deep convolutional neural network model could identify both ileocecal valve and appendiceal orifice

Patients	Number of frames with AO	Accuracy of detecting AO, %	Number of frames with ICV	Accuracy of detecting ICV, %
1	31	100	24	50
2	17	82.4	13	46.2
3	28	89.3	24	100
4	31	0	29	93.1
5	16	87.5	23	100
6	17	94.1	11	100
7	22	95.5	19	100
8	21	100	23	95.7
9	25	100	25	100
10	7	71.4	10	100
11	24	0	6	100
12	24	95.8	15	100
13	23	100	15	93.3
14	24	79.2	18	100
15	21	95.2	19	100
16	22	100	18	100
17	26	92.3	21	100
18	21	95.2	26	96.2
19	31	100	15	100
20	18	100	24	87.5
21	4	0	14	100

*AO*, appendiceal orifice; *ICV*, ileocecal valve; *CI*, confidence interval.

Both AO and ICV could concomitantly be detected in:

(1) 18 out of 21 patients (85.7%; 95% CI 63.7% to 97.0%) if accuracies were above threshold of 40%.

(2) 17 out of 21 patients (81.0%; 95% CI 58.1% to 94.6%) if accuracies were above threshold of 50%.

(3) 16 out of 21 patients (76.2%; 95% CI 52.8% to 91.8%) if accuracies were above threshold of 60%.

(4) 16 out of 21 patients (76.2%; 95% CI 52.8% to 91.8%) if accuracies were above threshold of 70%.

(5) 14 out of 21 patients (66.7%; 95% CI 43.0% to 85.4%) if accuracies were above threshold of 80%.

(6) 11 out of 21 patients (52.4%; 95% CI 29.8% to 74.3%) if accuracies were above threshold of 90%.

The accuracy of the model for differentiating AO, ICV, and polyps from normal mucosa was 86.4% (95% CI 84.1% to 88.5%), 86.4% (95% CI 84.1% to 88.6%), and 88.6% (95% CI 86.6% to 90.3%), respectively (Table 3). The accuracy of the model was 90.8% (95% CI 89.2% to 92.3%) for differentiating AO from ICV and normal mucosa, and 93.0% (95% CI 91.5% to 94.3%) for differentiating ICV from AO and normal mucosa. The per-patient accuracies are presented in the Supplementary file.

**Table 3.** Summary of the performance of the deep convolutional neural network artificial intelligence algorithm for the test data set

Detected landmarks	Total number of images	Number of TP	Number of TN	Number of FP	Number of FN	sensitivity (95% CI)	specificity (95% CI)	NPV (95% CI)	PPV (95% CI)	Accuracy (95% CI)	AUC (95% CI)
Normal mucosa vs. AO	983	381	468	62	72	84.1 (80.4 to 87.4)	88.3 (85.3 to 90.9)	86.7 (83.5 to 89.42)	86.0 (82.4 to 89.1)	86.4 (84.1 to 88.5)	90.8 (88.8 to 92.8)
Normal mucosa vs. ICV	922	345	452	78	47	88.0 (84.4 to 91.1)	85.3 (82.0 to 88.2)	90.58 (87.7 to 93.0)	81.6 (77.5 to 85.1)	86.4 (84.1 to 88.6)	94.4 (93.0 to 95.8)
Normal mucosa vs. polyp	1,172	566	472	58	76	88.2(85.4 to 90.6)	89.1(86.1 to 91.6)	86.1 (83.0 to 88.9)	90.7 (88.2 to 92.9)	88.6 (86.6 to 90.3)	94.8 (93.9 to 96.0)
Normal mucosa and ICV vs. AO^*^	1,375	372	877	45	81	82.1(78.3 to 85.5)	95.1 (93.5 to 96.4)	91.5 (89.6 to 93.2)	89.2 (85.8 to 92.0)	90.8 (89.2 to 92.3)	93.6 (92.2 to 95.0)
Normal mucosa and AO vs. ICV^*^	1,375	365	914	69	27	93.1 (90.1 to 95.4)	93.0(91.2 to 94.5)	97.1 (95.9 to 98.1)	84.1(80.3 to 87.4)	93.0 (91.5 to 94.3)	97.6 (96.8 to 98.3)
Normal mucosa, AO and ICV vs. polyp^*^	2,017	480	1,294	81	162	74.8 (71.2 to 78.1)	94.1 (92.7 to 95.3)	88.9 (87.2 to 90.4)	85.6 (82.4 to 88.4)	88.0 (86.5 to 89.3)	93.4 (92.3 to 94.5)
Normal mucosa, polyp and ICV vs. AO^*^	2,017	321	1,509	55	132	70.9 (66.4 to 75.0)	96.5 (95.5 to 97.3)	93.0 (90.5 to 93.23	85.4 (81.4 to 88.8)	90.7 (89.4 to 92.0)	95.3 (94.3 to 96.3)
Normal mucosa, polyp and AO vs. ICV^*^	2,017	343	1,502	123	49	87.5 (83.8 to 90.6)	92.4 (91.0 to 93.7)	96.84 95.84 to 97.7)	73.6 (69.4 to 77.6)	91.5 (90.2 to 92.7)	97.8 (97.3 to 98.3)

^*^The numbers were aggregated at the final step after getting results.

*TP/TN*, true positives/negatives; *FP/TN*, false positives/negatives; *NPV*, negative predictive value; *PPV*, positive predictive value; *AUC*, area under receiver operating characteristic curve; *CI*, confidence interval; *AO*, appendiceal orifice; *ICV*, ileocecal valve.

The false-positive rates of detecting AO, ICV, and polyp (vs. normal mucosa) were 11.7%, 14.7%, and 10.9%, respectively. The inference time of the model for each image frame was around 100 ms.

**Figure 2.**
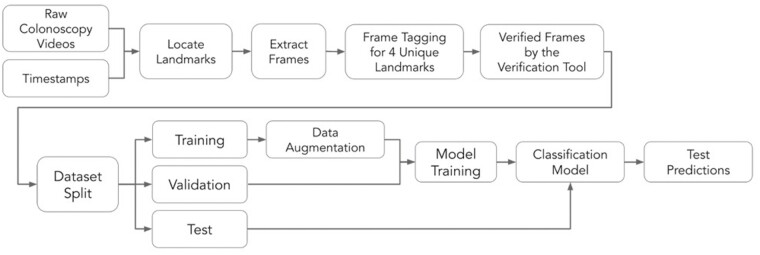
Illustration of data preparation, frame-by-frame landmark tagging, and quality assessment workflow for building disjoint databases for training and validation of a deep convolutional neural network classification model, and final prediction of landmarks in unseen test data.

Table 3 shows detailed results of the AI model performance in the test data set. Figure 2 shows the AUC of the AI algorithm for detecting each anatomical landmark in the test set.

## DISCUSSION

To the best of our knowledge, this study describes the first AI model to use a DCNN to automatically detect AO and ICV, and differentiate them from polyps and normal colon mucosa. Results showed that the model was able to differentiate these landmarks from polyps and normal mucosa with high accuracy. The model automatically detected both AO and ICV in 86% of patients in our test set. It also demonstrated a high ability (AUCs ≥ 90%) to distinguish AO, ICV, and polyps from normal mucosa in the test set. The required images for developing this model were prospectively obtained from a cohort of consecutive patients undergoing screening, surveillance, or diagnostic colonoscopies by multiple endoscopists, thus, enhancing generalizability, and reducing training, selection, and operator bias.

The U.S. MultiSociety Task Force on Colorectal Cancer suggests that visualization and documentation of the ICV and AO with photo-documentation is compulsory and an essential part of a high-quality colonoscopy (17). DCNN-based AI-assisted colonoscopy is a state-of-the-art system that already assists endoscopists with polyp detection and classification through commercially available solutions (18). Adding an AI module confirming completeness of a colonoscopy procedure seems a logical next step in the evolution of AI-assisted colonoscopy practice, as performing a complete colonoscopy is a vital prerequisite for a high ADR, and for minimizing the risk of interval cancer (3,19,20). Therefore, we aimed to create a model that can reliably detect both structures (e.g., AO and ICV) and distinguish them from normal mucosa and polyps. The combined detection of AO and ICV also avoids misreading of a diverticulum as confirmation of a complete colonoscopy.

Few studies have developed and tested new AI and nonAI approaches for identifying anatomical landmarks. These studies have the following major drawbacks: a small sample size, use of image-based data, low ADR, lack of testing in an independent data set, confusing alarm system, lack of DCNN technology, and never exceeding a prototype. One initial research used the non-AI K-mean classifier technique to automatically classify the 800 manually-annotated images derived from five colonoscopies into either appendix image or nonappendix image classes (21). Although the model accuracy was promising (90%), the exclusion of the images containing tangential AO and a relatively high false positive classification rate precluded further clinical application of the model. Likewise, Wang et al. used two nonAI algorithms to automatically detect AO (22). The initial algorithm distinguished images containing AO from others by analyzing geometric shape, saturation, and intensity changes along the edge’s cross-section. The second algorithm identified videos containing an appendix by analyzing frame intensity histograms to detect a near-camera pause during AO inspection. The average sensitivity and specificity of the first algorithm was 96.86% and 90.47%, respectively. The average accuracy of the second algorithm for detecting appendix videos was 91.30%. However, this study used only 23 colonoscopy videos and was not validated in an independent data set, which limits its generalizability. Recent advances in AI and deep learning have led to a growing consensus on the possibility of automatic detection of a complete colonoscopy. An AI model using CNN algorithm was developed using 3222 images extracted from 35 colonoscopy videos to detect the AO irrespective of bowel preparation (23). The accuracy and AUC of this model was 94% and 98%, respectively. However, this model has never been tested in practice. Another CNN model was trained using 6487 colon images prospectively obtained from over 300 colonoscopy procedures and annotated by two expert endoscopists for anatomic landmarks, lesions, and bowel preparation adequacy (24). This model intended to automatically calculate CIR and withdrawal time. The model accuracy was 88% when trained on all images including unprocessed and suboptimal-quality images, but increased to 98% accuracy and 99% AUC when trained on a subset of 1000 optimal images. The model’s effectiveness in real-time colonoscopy has remained untested. Furthermore, a study developed both image-based and video-based CNN models to calculate withdrawal time from the timepoint of detecting the ICV. The highest accuracy of 99.6% was achieved with an image-based data set, but only 70% accuracy was obtained with a video-based data set (25). Another recent study trained an AI algorithm using colonoscopy images (not obtained from a prospective patient cohort) to detect the AO, resulting in a 95% AUC in the test data set (26).

Our DCNN model could be integrated into colonoscopy reporting software. We imagine future applications that could automatically document landmark identification timepoints and generate automated reports postcolonoscopy, including all relevant procedural steps (identification time of ICV, AO, polyps), along with photo-documentation and withdrawal time calculations. Other potential applications include auditing tools. Previous attempts to develop and link auditing tools to real-time endoscopy practice have been challenging, mainly due to the significant administrative and budgetary burden placed on hospitals and the lack of structured endoscopic educational systems. To our knowledge, no auditing system has been designed and tested to provide simultaneous and automatic feedback on procedure quality and polyp classification as well as generate electronic reports. Our proposed model can be integrated into endoscopy practice as a didactic or practice audit system, used by experts and trainees, for providing a unified screening, intervention, and educational modality. Moreover, this system offers the potential to be coupled with the computer-assisted modules to obviate the bias raised by self-reporting and self-evaluation of practice quality.

The strengths of this study include the use of a large number of colonoscopy videos prospectively collected by multiple endoscopists, resulting in a mixture of colonoscopy findings (i.e., normal mucosa and polyps) and a high number of extracted frames. This model worked with unprocessed frames, and used the polyp images regardless of the polyp anatomical location and histology. Two experts reviewed all colonoscopy images, and a third expert endoscopist made the final annotation in cases of disagreement to ensure a high inter-rater agreement. The DCNN AI model is robust as it was trained end-to-end, resulting in performing classification tasks within the same learning model. Additionally, advanced equipment (i.e., high-definition endoscopes) were used for performing and recording all colonoscopies, following recommendations to use high-definition colonoscopes for screening and surveillance colonoscopy to effectively improve detection, resulting in high-quality videos and images.

However, the study does present some limitations. We included only colonoscopies of patients with adequate bowel preparation. As a result, it is necessary to further examine the generalizability of this model in real-time clinical application, ideally through a multicenter clinical trial using a higher number of colonoscopies. Furthermore, our model does not aim to distinguish anatomical landmarks from other lesions such as diverticula. Moreover, the total processing time was 100 ms, which is longer than the 33 ms of recommended inference time per frame for real-time system implication. Nonetheless, the strategies followed in this research for AI model training did not include advanced machine learning optimization and pruning techniques to decrease inference time. Further research should incorporate appropriate techniques to enhance model’s inference time and detection accuracy. Additionally, it is recommended to validate the model on a video-based data set to evaluate its performance in operational context.

To conclude, we developed a DCNN model that can reliably identify both AO and ICV in a test set of images from colonoscopy procedures. Furthermore, the DCNN model could distinguish AO and ICV from normal mucosa and colorectal polyps with high accuracy. We believe that this study is the first crucial step in creating a better automated colonoscopy reporting and auditing system that can deliver a colonoscopy report immediately after a procedure, including automated photo-documentation of anatomical landmarks and polyps.

## Supplementary Material

gwad017_suppl_Supplementary_Material

## Data Availability

The data that supports the findings of this study are available upon request from the corresponding author. The data are not publicly available due to privacy or ethical restrictions.
