# Supplementary material for: Automated Detection of Anatomical Landmarks During Colonoscopy Using a Deep Learning Model
Source: J Can Assoc Gastroenterol. 2023 May 2;6(4):145–51. doi: 10.1093/jcag/gwad017 (PMC10395661; doi:10.1093/jcag/gwad017)
Supplement: gwad017_suppl_Supplementary_Material [file gwad017_suppl_supplementary_material.docx]

**Supplementary Material**

**Exclusion Criteria**

Patients with active coagulopathy, inflammatory bowel diseases, familial polyposis syndrome, poor general health (defined as an American Society of Anesthesiologists [ASA] physical status class >3), need for emergency colonoscopies, or those who were hospitalized or in the emergency room were excluded from the study. Patients with inadequate bowel cleanliness, defined as a total Boston Bowel Preparation Score (BBPS) <6 or score <2 in the right segment,^1^ and those with a history of hemicolectomy were also excluded (n = 23).

**Supplementary Table 1.** Patients and procedures baseline characteristics

| **Variables** | **Training dataset**  **(n = 272)** | **Validation dataset**  **(n = 25)** | **Test dataset**  **(n = 21)** |
| --- | --- | --- | --- |
| **Age, median (IQR), years** | 64.0 (14.0) | 65.0 (8.5) | 67.0 (13.5) |
| **Sex, n (%)** |  |  |  |
| Male | 148 (54.4) | 15 (60.0) | 10 (47.6) |
| Female | 124 (45.6) | 10 (40.0) | 11 (52.4) |
| **Family history of CRC, n (%)** |  |  |  |
| No | 199 (73.2) | 18 (72.0) | 14 (66.7) |
| Yes | 56 (20.6) | 6 (24.0) | 7 (33.3) |
| Unknown | 17 (6.2) | 1 (4.0) | **-** |
| **Colonoscopy indication, n (%)** |  |  |  |
| Screening | 41 (15.1) | 2 (8.0) | 1 (4.8) |
| Positive FIT | 22 (8.1) | 2 (8.0) | 3 (14.3) |
| Adenoma surveillance | 128 (47.1) | 14 (56.0) | 9 (42.9) |
| CRC surveillance | 9 (3.3) | **-** | **-** |
| Anemia/bleeding | 31 (11.4) | 4 (16.0) | 5 (23.8) |
| Polypectomy | 7 (2.6) | - | **-** |
| Diarrhea | 5 (1.8) | 1 (4.0) | 2 (9.5) |
| Other | 29 (10.7) | 2 (8.0) | 1 (4.8) |
| **Endoscopy device, n (%)** |  |  |  |
| CF-HQ190L | 264 (97.1) | 25 (100) | 20 (95.2) |
| PCF-H190L | 4 (1.5) | **-** | **-** |
| Other | 4 (1.5) | **-** | 1 (4.8) |
| **Ileocecal valve identified, n (%)** |  |  |  |
| Yes | 263 (96.7) | 25 (100) | 21 (100) |
| **Appendiceal orifice identified, n (%)** |  |  |  |
| Yes | 257 (94.5) | 24 (96.0) | 21 (100) |
| **Withdrawal time, median (IQR), minutes** | 9.6 (7.8) | 8.4 (2.8) | 12.6 (7.8) |
| **Polyp detection rate, %** | 62.1 | 64.0 | 100 |
| **Number of identified polyps** | 473 | 26 | 41 |
| **Polyp size, median (IQR), mm** | 3.0 (4.0) | 3.0 (2.0) | 2.0 (2.5) |
| **Paris classification, n (%)** |  |  |  |
| IP | 30 (6.3) | **-** | 2 (4.9) |
| IS | 323 (68.3) | 23 (88.5) | 29 (70.7) |
| IIa | 41 (8.7) | 2 (7.7) | 7 (17.1) |
| IIb | - | **-** | - |
| IIc | 8 (1.7) | **-** | 3 (7.3) |
| III | - | **-** | - |
| **Pathology, n (%)** |  |  |  |
| Normal mucosa | 28 (5.9) | 1 (3.8) | 7 (17.1) |
| Hyperplastic | 76 (16.1) | 10 (38.5) | 11 (26.8) |
| Tubular adenoma | 212 (44.8) | 11 (42.3) | 16 (39.0) |
| Tubulovillous adenoma | 17 (3.6) | **-** | 3 (7.3) |
| Villous adenoma | 3 (0.6) | **-** | **-** |
| Traditional serrated adenoma | 2 (0.4) | **-** | - |
| Sessile serrated polyp/adenoma | 24 (5.1) | **-** | 2 (4.9) |
| High-grade dysplasia | 1 (0.2) | **-** | 1 (2.4) |
| Other | 13 (2.7) | **-** | 1 (2.4) |
| Not retrieved | 31 (6.6) | **-** | **-** |
| Missing | 66 (14.0) | 1 (3.8) | **-** |

*IQR*, interquartile range; *CRC*, colorectal cancer; *FIT*, fecal immunologic test.

Per-patient accuracy of the deep convolutional neural network artificial intelligence algorithm for the test dataset. All values are presented as percentage (%). (TP: true positive; FP: false positive; CI: confidence interval; AO: appendiceal orifice; ICV: ileocecal valve)

**AO vs normal mucosa:**

Threshold: 10.0, TP: 21, FP: 0, Accuracy: 100.0, 95% CI: 83.89 to 100

Threshold: 20.0, TP: 21, FP: 0, Accuracy: 100.0, 95% CI: 83.89 to 100

Threshold: 30.0, TP: 21, FP: 0, Accuracy: 100.0, 95% CI: 83.89 to 100

Threshold: 40.0, TP: 20, FP: 1, Accuracy: 95.24, 95% CI: 76.18 to 99.88

Threshold: 50.0, TP: 20, FP: 1, Accuracy: 95.24, 95% CI: 76.18 to 99.88

Threshold: 60.0, TP: 19, FP: 2, Accuracy: 90.48, 95% CI: 69.62 to 98.83

Threshold: 70.0, TP: 18, FP: 3, Accuracy: 85.71, 95% CI: 63.66 to 96.95

Threshold: 80.0, TP: 17, FP: 4, Accuracy: 80.95, 95% CI: 58.09 to 94.55

Threshold: 90.0, TP: 13, FP: 8, Accuracy: 61.9, 95% CI: 38.44 to 81.89

-----------------------------------------------

**ICV vs normal mucosa:**

Threshold: 10.0, TP: 21, FP: 0, Accuracy: 100.0, 95% CI: 83.89 to 100

Threshold: 20.0, TP: 21, FP: 0, Accuracy: 100.0, 95% CI: 83.89 to 100

Threshold: 30.0, TP: 21, FP: 0, Accuracy: 100.0, 95% CI: 83.89 to 100

Threshold: 40.0, TP: 21, FP: 0, Accuracy: 100.0, 95% CI: 83.89 to 100

Threshold: 50.0, TP: 20, FP: 1, Accuracy: 95.24, 95% CI: 76.18 to 99.88

Threshold: 60.0, TP: 19, FP: 2, Accuracy: 90.48, 95% CI: 69.62 to 98.83

Threshold: 70.0, TP: 17, FP: 4, Accuracy: 80.95, 95% CI: 58.09 to 94.55

Threshold: 80.0, TP: 17, FP: 4, Accuracy: 80.95, 95% CI: 58.09 to 94.55

Threshold: 90.0, TP: 12, FP: 9, Accuracy: 57.14, 95% CI: 34.02 to 78.18

-----------------------------------------------

**Polyp vs normal mucosa:**

Threshold: 10.0, TP: 21, FP: 0, Accuracy: 100.0, 95% CI: 83.89 to 100

Threshold: 20.0, TP: 21, FP: 0, Accuracy: 100.0, 95% CI: 83.89 to 100

Threshold: 30.0, TP: 21, FP: 0, Accuracy: 100.0, 95% CI: 83.89 to 100

Threshold: 40.0, TP: 21, FP: 0, Accuracy: 100.0, 95% CI: 83.89 to 100

Threshold: 50.0, TP: 20, FP: 1, Accuracy: 95.24, 95% CI: 76.18 to 99.88

Threshold: 60.0, TP: 20, FP: 1, Accuracy: 95.24, 95% CI: 76.18 to 99.88

Threshold: 70.0, TP: 19, FP: 2, Accuracy: 90.48, 95% CI: 69.62 to 98.83

Threshold: 80.0, TP: 18, FP: 3, Accuracy: 85.71, 95% CI: 63.66 to 96.95

Threshold: 90.0, TP: 14, FP: 7, Accuracy: 66.67, 95% CI: 43.03 to 85.41

-----------------------------------------------

**Normal mucosa+ICV vs AO:**

Threshold: 10.0, TP: 21, FP: 0, Accuracy: 100.0, 95% CI: 83.89 to 100

Threshold: 20.0, TP: 21, FP: 0, Accuracy: 100.0, 95% CI: 83.89 to 100

Threshold: 30.0, TP: 21, FP: 0, Accuracy: 100.0, 95% CI: 83.89 to 100

Threshold: 40.0, TP: 21, FP: 0, Accuracy: 100.0, 95% CI: 83.89 to 100

Threshold: 50.0, TP: 21, FP: 0, Accuracy: 100.0, 95% CI: 83.89 to 100

Threshold: 60.0, TP: 20, FP: 1, Accuracy: 95.24, 95% CI: 76.18 to 99.88

Threshold: 70.0, TP: 19, FP: 2, Accuracy: 90.48, 95% CI: 69.62 to 98.83

Threshold: 80.0, TP: 18, FP: 3, Accuracy: 85.71, 95% CI: 63.66 to 96.95

Threshold: 90.0, TP: 16, FP: 5, Accuracy: 76.19, 95% CI: 52.83 to 91.78

-----------------------------------------------

**Normal mucosa+AO vs ICV:**

Threshold: 10.0, TP: 21, FP: 0, Accuracy: 100.0, 95% CI: 83.89 to 100

Threshold: 20.0, TP: 21, FP: 0, Accuracy: 100.0, 95% CI: 83.89 to 100

Threshold: 30.0, TP: 21, FP: 0, Accuracy: 100.0, 95% CI: 83.89 to 100

Threshold: 40.0, TP: 21, FP: 0, Accuracy: 100.0, 95% CI: 83.89 to 100

Threshold: 50.0, TP: 21, FP: 0, Accuracy: 100.0, 95% CI: 83.89 to 100

Threshold: 60.0, TP: 21, FP: 0, Accuracy: 100.0, 95% CI: 83.89 to 100

Threshold: 70.0, TP: 21, FP: 0, Accuracy: 100.0, 95% CI: 83.89 to 100

Threshold: 80.0, TP: 18, FP: 3, Accuracy: 85.71, 95% CI: 63.66 to 96.95

Threshold: 90.0, TP: 16, FP: 5, Accuracy: 76.19, 95% CI: 52.83 to 91.78

1. Kastenberg D, Bertiger G, Brogadir S. Bowel preparation quality scales for colonoscopy. *World J Gastroenterol* 2018; **24**(26): 2833-43.
